# Parsonage-Turner Syndrome and Occupational Therapy Interventions: A Case Report

**DOI:** 10.7759/cureus.76511

**Published:** 2024-12-28

**Authors:** Stephanie Roberts, Abigail Skarbinski, Jeanine Beasley, Ashley Brokenshaw, Tara Fedewa, Terese Feldpausch, Hanna Hulings, Michael J Shoemaker, Claire Dolislager

**Affiliations:** 1 Department of Occupational Therapy, ClearSky Rehabilitation Hospital, Weatherford, USA; 2 Department of Occupational Therapy, Grand Valley State University, Grand Rapids, USA; 3 Department of Physical Therapy, Grand Valley State University, Grand Rapids, USA

**Keywords:** activities of daily living, brachial plexus neuritis, hemiplegia, neuralgic amyotrophy, occupational therapy, shoulder pain

## Abstract

Parsonage-Turner syndrome (PTS) is a rare brachial plexus neuropathy with a sudden onset of upper extremity pain, weakness, and loss of range of motion (ROM). Studies on occupational therapy (OT) interventions are limited. The aim of this case report was to explore the OT experiences, interventions, and outcomes of a patient with PTS. The patient was a 44-year-old woman with COVID-19 who was admitted to the intensive care unit (ICU). She was intubated and placed in the prone position for 16 hours a day. The diagnosis of PTS was confirmed. Data included the OT medical record and interviews with the patient and the treating occupational therapist. The OT intervention type and frequency are reported. Outcome measurements showed 80-100% improvement for the affected left upper extremity (LUE). Manual muscle testing scores returned to WNL except for shoulder internal and external rotation, with improved but limited grip, pinch, and fine motor coordination. The qualitative data emphasized the importance of considering the sociocultural perspective and psychosocial effects of PTS with OT interventions. This study reported the OT interventions and outcomes of this rare condition. All outcome measures demonstrated improvement, many within normal limits. Considering the sociocultural and psychosocial effects of the patient appeared to contribute to improved outcomes.

## Introduction

Parsonage-Turner syndrome (PTS) is a rare condition affecting two to three individuals out of every 100,000 [1]. It is characterized by upper extremity weakness, decreased shoulder range of motion, and shoulder pain [2-4] with debilitating atrophy of the upper extremity [5,6]. PTS was first described by Dreschfeld in 1887 [7]; however, further description was reported by Parsonage and Turner in 1948 [8-10]. Since then, the condition has been known by many different names, such as brachial plexitis, neuralgic amyotrophy (NA), acute brachial neuropathy, acute brachial plexitis, idiopathic brachial plexopathy, idiopathic brachial neuritis, paralytic brachial neuritis, and brachial, radiculitis, among others [9,11,12]. Neuropathic pain at onset and patchiness of attacks are distinct features of PTS [4,11]. Acute motor axon loss results in symptoms such as burning and tingling sensations in the shoulder, glenohumeral joint pain, and absence of sensory responses [13-17].

The etiology of PTS remains unclear [12,14,18-20]. PTS has, however, been found to affect individuals who have had shoulder surgery or have had diseases such as Lyme disease [2,6]. A study by Mitry et al. in 2020 found the most common pathophysiological causes of PTS to be viral illness (25%) and recent immunization (15%) [14]. Autoimmune abnormalities, genetic factors, and trauma have been associated with the onset of PTS [8,10,11,14,19]. A study by Alvarado et al. in 2021 reported a diagnosis of PTS following COVID-19 infection and hypothesized that the infection triggered an immune-mediated reaction involving the brachial plexus [13]. Sensory symptoms occur in 42%-78% of cases but typically do not match the areas of pain and paresis [14,18]. Trophic skin changes, edema, temperature dysregulation, changes in hair or nail growth, or sweating may also occur [18]. Complement-fixing antibodies to peripheral nerve myelin increase in the acute phase of PTS but decrease in the recovery phase [18].

This case is unique in that there have been limited studies on the effectiveness of specific OT interventions for patients with PTS [1]. However, some studies have demonstrated that occupational and physical therapy interventions, when focused on activities of daily living (ADL), resulted in improvements in upper limb strength, active range of motion (AROM), and functional capability in patients with PTS [21,22]. In a study by Ijspeert et al., function and satisfaction improved with physical and occupational therapy interventions, yet patients reported persistent fatigue [1]. A case study by Ibrahim et al. reported decreased sensation due to the involvement of cervical spinal nerves [2]. A study by Feinberg et al. reported peripheral nerve growth approximately six months after onset, increased muscle strength at ten months, and complete recovery at one year [8].

This case report aims to contribute to identifying the role of OT and possible OT interventions for patients with PTS by presenting the OT interventions, experiences, and outcomes of an individual with PTS. This article was previously presented as a poster at the 2024 annual American Occupational Therapy Association meeting in Orlando, Florida [23].

## Case presentation

Patient information

The patient was a 44-year-old woman with a noted past medical history of Hashimoto’s thyroiditis diagnosed with COVID-19 and admitted into the intensive care unit (ICU). She was intubated two days later and placed in the prone position for 16 hours per day for eight days. The patient noticed a sudden onset of left upper extremity (LUE) pain and hemiplegia around the time of extubation, and OT intervention was initiated. Imaging of the LUE showed low-grade partial-thickness tears of supraspinatus and infraspinatus, low-grade partial-thickness delamination tear of the supraspinatus tendon, bursitis at the subacromial subdeltoid bursa, a small glenohumeral effusion, shoulder atrophy, and an intact brachial plexus. Following nerve conduction testing completed by a neurologist, the patient received a confirmed diagnosis of PTS and was prescribed gabapentin. The patient additionally consulted with a physiatrist to confirm this diagnosis. The patient was discharged to a rehabilitation facility for one week prior to returning home. Outpatient OT continued twice a week to address the LUE pain and hemiplegia.

OT interventions included positioning, modalities, neurological and occupation-based exercises, and activities. As shown in Table 1, in the first three months of outpatient OT (sessions 1-20), the interventions used most frequently included proprioceptive neuromuscular facilitation (PNF) patterns, kinesiotape to stabilize the shoulder and decompress the wrist, LUE AROM exercises, and neuromuscular retraining in a gravity-eliminated plane, progressing to against gravity. Tables 2, 3 show the last five months of therapy (sessions 21-42), the interventions progressed to resistive exercises, movement training, weighted activities, and neuromuscular retraining. These interventions focused on muscle strengthening and coordination with the return of AROM.

**Table 1:** Timeline: sessions 1-20 intervention frequency. PNF: proprioceptive neuromuscular facilitation, LUE: left upper extremity, AROM: active range of motion, BUE: bilateral upper extremities, NMES: neuromuscular electrical stimulation, OT: occupational therapy.

OT intervention	Times provided in 19 sessions	Percent of sessions (%)
PNF patterns	13	68
Kinesiotape	11	58
LUE AROM	11	58
Chest press LUE unweighted	9	47
Dowel exercises BUE	6	32
Towel slides	6	32
UE ergometer	6	32
NMES LUE	5	26
Pulleys	5	26
Russian stimulation	4	21
Free weight chest press	4	21
Free weight shoulder flexion	4	21
Weightbearing	3	16
TheraBand	3	16
Hand strengthening grasp/release	3	16
FlexBar–horizontal wringing, vertical wringing, pronation/supination	2	11
Spring loaded gripper	2	11
Rolling medicine ball	2	11
Bicep curl	2	11
Hammer curl	2	11
Reverse curl	2	11
Grasping and reaching activity	2	11
Occupation-based re-education	2	11

**Table 2:** Timeline: session 21-42 intervention frequency proximal muscles. OT: occupational therapy, PNF: proprioceptive neuromuscular facilitation, LUE: left upper extremity, AROM: active range of motion.

OT Intervention	Times provided in 21 sessions	Percent of sessions (%)
Bicep curl	19	90
Hammer curl	18	86
Free weight chest press	15	71
Free weight shoulder flexion	11	52
Free weight shoulder abduction	11	52
Kinesiotape	10	48
Free weight internal/external rotation	10	48
Free weight overhead press	10	48
Weighted PNF	9	43
Free weight overhead tricep extension	9	43
Reverse curl	7	33
LUE AROM	4	19

**Table 3:** Timeline: session 21-42 intervention frequency distal muscles. OT: occupational therapy.

OT intervention	Times provided in 19 sessions	Percent of sessions (%)
Free weight pronation/supination	10	48
FlexBar-horizontal wringing, vertical wringing, pronation/supination	9	43
Hand strengthening grasp release	7	33
Ball catch and chest pass	7	33
Fine motor activities	7	33
Free weight ulnar deviation and radial deviation	5	24
Grasping and reaching activity	4	19
Free weight wrist flexion and extension	4	19

Methods

IRB approval was obtained (Protocol #23-256-H), and the patient signed a release of medical information form. The patient and the treating clinician also completed informed consent forms. Data extracted from the outpatient OT medical record included demographics, frequency and type of interventions, and outcome measures. Descriptive statistics were completed.

**Figure 1:**
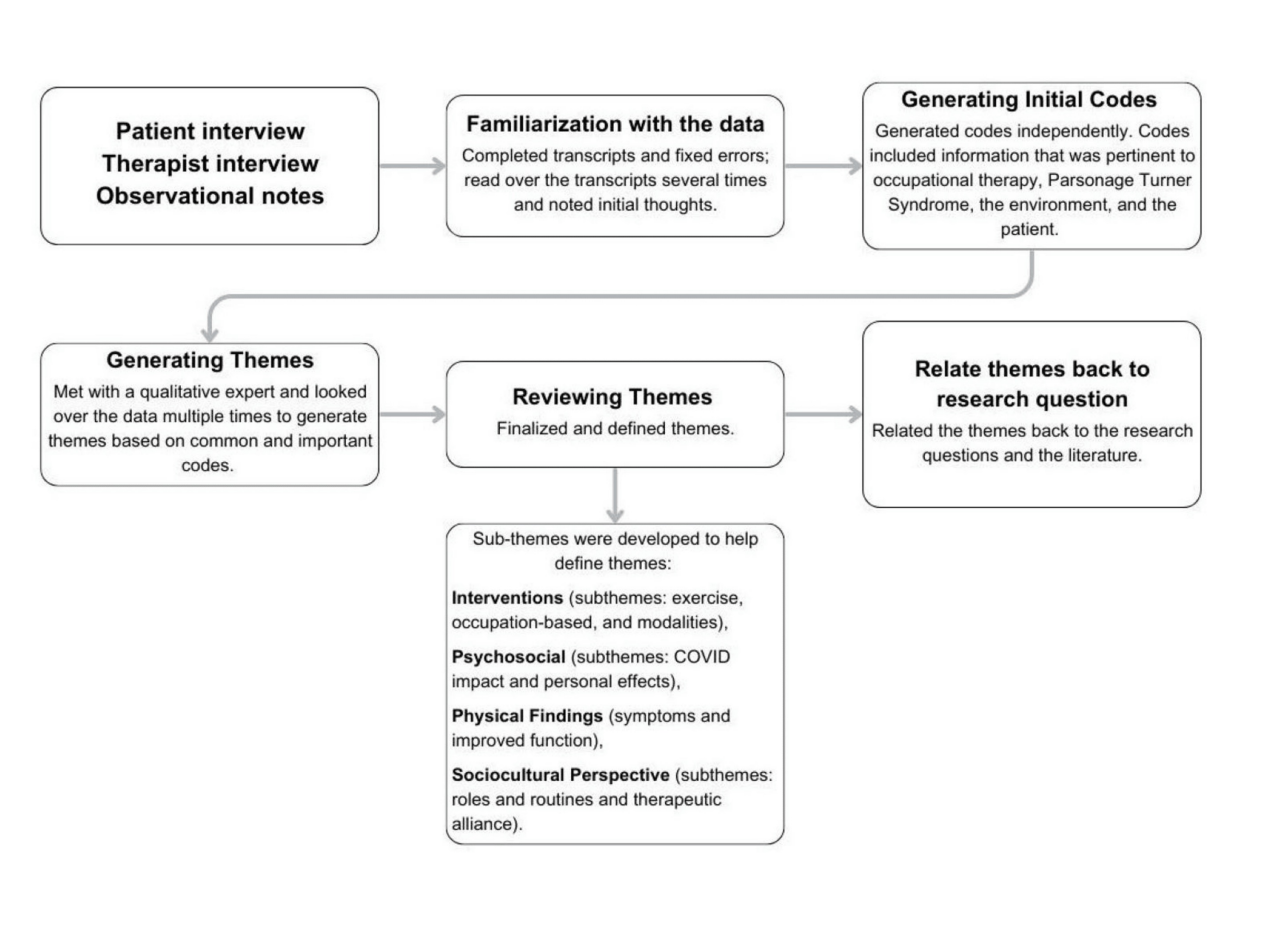
Qualitative analysis. Qualitative analysis is based on the steps by Braun and Clarke [26].

The patient was interviewed and recorded with open-ended questions adapted from Brazier et al. [24]. The treating occupational therapist was asked open-ended questions based on the Occupational Therapy Practice Framework: Domain and Process [25]. The qualitative data was analyzed in six phases, as outlined by Braun and Clarke [26], to determine themes (Figure 1).

Clinical findings

Quantitative Data: Outcome Measures

Outcome measures included disabilities of the shoulder, arm, and hand (QuickDASH), manual muscle testing (MMT), grip strength, pinch strength, and the Nine Hole Peg Test. After the patient’s initial evaluation, the patient was re-evaluated six times throughout the course of treatment. Table 4 reports the initial evaluation scores, final evaluation scores, and the percentage of improvement.

**Table 4:** Outcome measurement results. *Indication of outcome measure within normal limits (WNL). MMT: manual muscle testing, LUE: left upper extremity, ER: external rotation, IR: internal rotation.

Outcome measure	Initial evaluation	Final evaluation	Change	Improvement (%)
QuickDASH	70.4	13.64	56.76	80
L shoulder MMT-flexion	1	5*	4	80
L shoulder MMT-extension	3	5*	2	40
L shoulder MMT-abduction	1	5*	4	80
L shoulder MMT-adduction	3	5*	2	40
L shoulder MMT-shoulder ER at 90 abduction	1	4	3	75
L shoulder MMT-shoulder IR at 90 abduction	1	4	3	75
L elbow MMT-flexion	1	5*	4	80
L elbow MMT-extension	3	5*	2	40
L elbow MMT-supination	2	5*	3	60
L elbow MMT-pronation	1	5*	4	80
L wrist MMT-flexion	3	5*	2	40
L wrist MMT-extension	2	5*	3	60
L power grip PSI	5	33	28	84
L lateral pinch PSI	7	14	7	50
L pincer PSI	4	6	2	33
L tripod pinch PSI	5	8	3	37
Nine Hole Peg test LUE in seconds	179	30	149	83

All patient outcome measures demonstrated improvement. The patient’s QuickDASH score showed a significant improvement of 56.76 points, with the minimally clinically important difference (MCID) requiring an improvement of at least 15.91 points [27]. The patient demonstrated LUE MMT within normal limits (WNLs) except for shoulder internal and external rotation, which was 4/5.

With the Nine Hole Peg Test, the patient achieved 30 seconds (norm 17.64 seconds) [28]. LUE grip and pinch strength improved but did not achieve norms for the patient’s age group and gender at discharge. Grip strength measured 33 lbs. (norm 61.7-62.3 lbs.) [29,30], but this was an 84% improvement that exceeded the MCID of 19.5% [31]. At discharge, the tip pinch was 6 lbs. (norm 11.1 lbs.), lateral/key pinch was 14 lbs. (norm 15.8 lbs.), and palmer/tripod pinch was 8 lbs. (norm 16.6lbs.) [30].

Qualitative Data: Overview

Thematic analysis of the patient and therapist interviews, as well as observational notes from the interviews, resulted in the discovery of four themes and nine subthemes: interventions (subthemes: exercise, occupation-based, and modalities), psychosocial (subthemes: COVID impact and personal effects), physical findings (symptoms and improved function), and sociocultural perspective (subthemes: roles and routines and therapeutic alliance).

Qualitative Data: Interventions

The therapist emphasized the use of exercise, occupation-based activities, and modalities. The occupational therapist described exercise progression as beginning in the supine with facilitation and neuromuscular retraining techniques such as weight-bearing and positioning. As AROM returned, the intervention progressed to against partial gravity with an active assist in sitting, then standing against gravity. Weighted activity was not added until AROM was achieved.

The patient described techniques learned in therapy to help with daily occupations, including compensatory strategies with the use of her left hand as a stabilizer and facilitator to assist with bimanual tasks. Modalities were also mentioned, including kinesiotape and transcutaneous electrical nerve stimulation (TENS) to alleviate pain and support the left shoulder and wrist.

Qualitative Data: Psychosocial

The patient reported prolonged prone positioning and lack of thorough bathing during her ICU stay caused rashes, skin infections, and scarring. The change in physical appearance, including weight loss and sudden loss of LUE function, negatively affected her self-image and self-esteem, such as feeling embarrassed to wear her sling at her children’s sporting events. The patient reported that COVID-19 also resulted in fatigue, stress, and brain fog, which limited her functional capacity in occupational roles.

Qualitative Data: Physical Findings

Physical findings were categorized into two subthemes: symptoms and improved function. LUE function and motor control improved distally to proximally, with exercises progressing from gravity-eliminated to against gravity to against resistance. The patient reported a decrease in the need for medications due to decreased pain with OT interventions. The patient noted persistent mild thumb pain and decreased left grip and pinch strength after discharge.

Qualitative Data: Sociocultural Perspective

Social, cultural, and environmental factors seemed to influence the patient’s experience with PTS and her engagement in OT. The patient and therapist emphasized the effects of interrupted roles and routines and a strong therapeutic alliance on rehabilitation. It became clear that the patient’s hard-working nature and passion for her roles both as a mother and as a nurse were strong motivators, and the therapist chose occupation-based interventions to address the patient’s deficits as related to her roles and values. The therapist attributed the patient’s OT attendance and home exercise program adherence to her embodiment of these roles.

## Discussion

This case report explored OT interventions for PTS, patient and therapist experiences, and patient outcomes. OT interventions included preparatory activities such as exercise, modalities, and occupation-based activities. Our case report agrees with the study by Labrecque emphasizing the importance of occupational therapists not only incorporating physical rehabilitation of the upper extremity, but also addressing the psychological effects of PTS through the therapeutic use of self, consideration of the impact of PTS on self-esteem and lifestyle, and client-centered goal setting [32]. In contrast, the subject of that case study had significant edema that was effectively decreased through the use of a compression glove [32]. Our case report agrees with the interventions described by Malik et al., including the use of TENS for an individual who was believed to have PTS after sudden onset of shoulder weakness and decreased AROM prior to discharge from a skilled nursing facility to inpatient rehabilitation [33]. In contrast, the individual in the Malik et al. [33] study was only treated in an inpatient rehabilitation setting, whereas the patient in this case report received one week of inpatient rehabilitation followed by 42 sessions of outpatient occupational therapy.

The main limitations of this case report are the sample size of only one patient with PTS and the non-experimental design, preventing the generalization of results to a population. Future research is recommended with larger sample sizes and an experimental design to determine the efficacy of specific OT interventions and establish treatment protocols.

## Conclusions

This study reported the types and frequencies of OT interventions and outcomes for this rare condition. MMT returned to WNL except for shoulder internal and external rotation with improved but limited grip, pinch, and fine motor coordination. This case report also emphasized the importance of considering the sociocultural perspective and psychosocial effects of the patient, which appeared to contribute to improved outcomes. All outcome measures demonstrated improvement, many within normal limits. These OT interventions positively impacted the patient’s performance and participation in activities of daily living, allowing the patient to return to the roles and routines she valued.
